# Comment on: An improved algorithm to screen for carbapenemase production in *Pseudomonas aeruginosa*

**DOI:** 10.1093/jacamr/dlag125

**Published:** 2026-07-02

**Authors:** Christian M Gill, David P Nicolau

**Affiliations:** Department of Clinical Pharmacy, Virtua Health, 100 Bowman Dr, Voorhees, NJ 08043, USA; Center for Anti-Infective Research and Development, Hartford Hospital, Hartford, CT, USA; Innoviva Specialty Therapeutics, Waltham, MA, USA

To the Editor,

We read with great interest Ellem and colleagues’ assessment of phenotypic screening criteria to guide carbapenemase testing in a reference set of carbapenem-resistant *Pseudomonas aeruginosa* (CRPA) isolates.^1^ We agree that continued iterations of screening algorithms are needed, particularly in a variety of geographic settings and inclusive of diverse carbapenemases. There are several points we would like to highlight considering this work.

The use of aminoglycoside resistance is logical due to the propensity of this mechanism to be co-harboured with carbapenemases on plasmids. The utility of tobramycin in this setting has been reported in two other cohorts of isolates that encompassed largely VIM-producing *P. aeruginosa* from Greece and the Netherlands. In these cohorts, only 2/80 and 3/130 carbapenemase producing isolates were susceptible to tobramycin, respectively.^2,3^ However, plasmid configuration may differ across geographic regions and carbapenemase enzyme subtypes. Similarly, there may be differences in laboratory procedures for aminoglycoside reporting where tobramycin may not be available. In a large multicentre study of CRPA that included isolates from the USA, China, South-Central America, the Middle East and Australia-Singapore, 40% and 20% of carbapenemase producing CRPA remained susceptible to amikacin and gentamicin, respectively.^4^ Amikacin’s enhanced activity relative to gentamicin or tobramycin is not unexpected as amikacin remains active in the face of some aminoglycoside resistance determinants. The differences in aminoglycoside potency must be considered relative to variability in standard testing capabilities of different laboratories as reports of amikacin susceptibility differ widely among surveillance studies. In a multinational analysis of CRPA, 40% of IMP-producing *P. aeruginosa* remained susceptible to amikacin compared with 16% of VIM-producing strains.^5^ Similarly, among *P. aeruginosa* harbouring KPC 75% of isolates tested as susceptible to amikacin, although tobramycin susceptibility was not reported in either study.^6^ Although tobramycin may be a more reliable surrogate for carbapenemase testing in this current assessment, robust validation across different carbapenemase classes and geographic regions is warranted.

We clarify the discussion from our previous work. The authors incorrectly reported our previous findings. In their reference 17, they suggest that the GES-harbouring strain was susceptible to ceftolozane/tazobactam, thus meaning it was missed by the criteria. As outlined in their table 2 and the text, the addition of ceftolozane/tazobactam-R criteria captured all six genotypically positive carbapenemase producers including five VIM-positive isolates and one GES-positive isolate.^7^ The only isolate missed by this algorithm was an mCIM positive isolate harbouring OXA-2 and OXA-50, which has been previously associated with falsely positive mCIM results.^8,9^ When we made further iterations on our initial phenotypic algorithm in the authors’ reference 27, the authors incorrectly stated that 13 GES-positive strains were missed by the algorithm including ceftolozane/tazobactam-non-susceptibility. This was also incorrect as, when the ceftolozane/tazobactam-based criterion was added, this reduced the number of carbapenemase producers missed by the base algorithm (imipenem or meropenem-R plus cefepime and ceftazidime-NS) including capturing all GES-harbouring strains but one. These studies highlight the utility of ceftolozane/tazobactam-non-susceptibility criteria to guide carbapenemase testing since, mechanistically, most carbapenemases readily hydrolyse ceftolozane/tazobactam.^8^ Similar criteria have been validated by other investigators.^10,11^ These data also highlight that a significant number of GES-carbapenemase and ESBL harbouring isolates are ceftolozane/tazobactam-resistant^12–15^ demonstrating that rapid detection of GES can aid clinicians in selecting optimal therapy since ceftolozane/tazobactam represents recommended therapy for CRPA.^16^

GES represent a diverse and underappreciated family of β-lactamases. Although many subtypes were initially considered ESBLs, single amino acid substitutions can alter the hydrolytic spectrum to include carbapenems.^17^ Although *P. aeruginosa* harbouring GES are increasingly recognized, the clinical implications of these enzymes are poorly understood. A single-centre analysis from Spain found that infections caused by ST-235 *P. aeruginosa* harbouring GES-5 and *exo*U virulence factor were associated with decreased survival compared with non-carbapenemase producers and VIM-2 carbapenemase producers.^12^ Despite this, there are limited clinical data to support optimal therapy. *In vitro* data have shown a high proportion of GES-harbouring *P. aeruginosa* encompassing both ESBL and carbapenemase subtypes are susceptible to ceftazidime/avibactam and cefiderocol, although clinical data validating the *in vitro* activity are sparse.^15,18,19^

When clinical data are unavailable, clinicians must rely on translational models to validate whether the *in vitro* activity noted is expected to produce sufficient microbiologic activity. Using the murine thigh infection model and antimicrobial exposures mimicking those seen in humans, ceftazidime/avibactam and cefiderocol reached the translational endpoint of >1-log_10_ kill against susceptible *P. aeruginosa* harbouring GES (both ESBL and carbapenemase subtypes).^19^ Ceftazidime/avibactam activity was more variable in some subtypes, including isolates harbouring tandem GES-alleles (ESBL plus ESBL or ESBL plus carbapenemase subtypes), consistent with the elevated MICs observed. Isolates harbouring various GES-subtypes resistant to ceftazidime/avibactam have been reported clinically. Indeed, a case report described a single amino acid substitution in the GES-5 (carbapenemase) during ceftazidime/avibactam therapy. The subsequent isolate harboured GES-15 (ESBL) with elevated MICs to the combination, however; the MICs to meropenem were reduced. This led to pre-clinical investigation assessing the utility of combining ceftazidime/avibactam and meropenem, which showed marked microbiologic activity *in vivo* using human simulated exposures.^20^ Integrating these translational *in vivo* findings with the *in vitro* MIC distributions provides rationale for building treatment algorithms in response to GES detection in clinical specimens. It is important to acknowledge that, particularly in the case of *P. aeruginosa*, these enzymes are present in the context of all the other resistance mechanisms inherent to these organisms. Thus, only considering traditional hydrolytic spectrum of the specific enzyme subtype may be misleading. For example, clinicians often associate ESBL production with the retained utility of carbapenems.^16^ However, in the context of GES and *P. aeruginosa*, the constellation of enzyme expression and other non-enzymatic resistance commonly leads to carbapenem-resistance.^21,22^ Studies should continue to identify common phenotypic patterns to guide clinical decision making by determining the most likely active therapy when GES-enzymes are detected in clinical isolates regardless of the traditional hydrolytic spectrum. More data are needed to further define the epidemiology and optimal treatment as GES-detecting assays are more broadly implemented.

As the diversity and prevalence of carbapenemases expressed in *P. aeruginosa* continues to evolve, validation and implementation of phenotypic criteria to guide carbapenemase testing are paramount to optimize the value of testing, define local epidemiology and guide antimicrobial selection. Such data are particularly needed for GES-harbouring strains to define optimal therapy.
